# Dicer-Dependent Biogenesis of Small RNAs Derived from 7SL RNA

**DOI:** 10.1371/journal.pone.0040705

**Published:** 2012-07-12

**Authors:** Yong-Feng Ren, Guiling Li, Jianmin Wu, Yong-Feng Xue, Yi-Jiang Song, Lu Lv, Xue-Jiao Zhang, Kai-Fu Tang

**Affiliations:** Institute of Genomic Medicine, Wenzhou Medical College, Wenzhou, People’s Republic of China; Beckman Research Institute of the City of Hope, United States of America

## Abstract

It has been reported that decreased Dicer expression leads to Alu RNAs accumulation in human retinal pigmented epithelium cells, and Dicer may process the endogenous SINE/B1 RNAs (the rodent equivalent of the primate Alu RNAs) into small interfering RNAs (siRNAs). In this study, we aimed to address whether Dicer can process Alu RNAs and their common ancestor, 7SL RNA. Using Solexa sequencing technology, we showed that Alu-derived small RNAs accounted for 0.6% of the total cellular small RNAs in HepG2.2.15 cells, and the abundance decreased when Dicer was knocked down. However, Alu-derived small RNAs showed different characteristics from miRNAs and siRNAs, the classic Dicer-processed products. Interestingly, we found that small RNAs derived from 7SL RNA accounted for 3.1% of the total cellular small RNAs in the control cells, and the abundance dropped about 3.4 folds in Dicer knockdown cells. Dicer-dependent biogenesis of 7SL RNA-derived small RNAs was validated by northern blotting. *In vitro* cleavage assay using the recombinant human Dicer protein also showed that synthetic 7SL RNA was processed by Dicer into fragments of different lengths. Further functional analysis suggested that 7SL RNA-derived small RNAs do not function like miRNAs, neither do they regulate the expression of 7SL RNA. In conclusion, the current study demonstrated that Dicer can process 7SL RNA, however, the biological significance remains to be elucidated.

## Introduction

During the past 65 million years, Alu elements have evolved to more than one million copies in the primate genomes. This makes them the most successful transposable elements in terms of copy number [1], [2]. The typical Alu element is ∼300 base pairs long and exhibits a dimeric structure, which is separated by an A-rich linker region. It contains an internal RNA polymerase III (Pol III) promoter (A and B boxes) at the 5′ region and ends with an oligo dA-rich tail of variable length [1]. Although being considered by some scientists as an example of “junk DNA”, Alu elements do play important roles. They affect the genome in several ways, causing insertion mutations, DNA recombination, gene conversion and altered gene transcription [1], [2]. In addition, Alu RNAs play major roles in post transcriptional regulation of gene expression, by affecting protein translation, alternative splicing and mRNA stability [3].

Alu elements can be transcribed by two independent polymerases in two different ways. “Free Alu RNAs” are transcribed by Pol III from their own promoters, while “embedded Alu RNAs” are transcribed by RNA polymerase II (Pol II) as part of protein- or non-protein-coding RNAs [3]. Some embedded Alu RNAs are transcribed by Pol II in the antisense direction, and may form bimolecular double-stranded RNAs (dsRNAs) with the sense Alu RNAs. Transcriptional read-through of inverted Alu elements may also form intramolecular dsRNAs [3]. Theoretically, these dsRNAs can be processed by double-stranded endonucleases. As a typical endonuclease, Dicer can process dsRNAs into small interfering RNAs (siRNAs) [4], [5]. It is also essential for the biogenesis of miRNAs via processing their precursors [5], [6]. Recently, Kaneko and colleagues reported that decreased Dicer expression leads to Alu RNAs accumulation in human retinal pigmented epithelium cells, and that Dicer can degrade synthetic Alu dsRNAs *in vitro* [7]. Although endogenous siRNAs derived from SINE/B1 RNAs, the rodent equivalent of the primate Alu RNAs [8], have been identified [9], [10], [11], [12], [13], direct evidence of endogenous Alu-derived siRNAs in human cells is still missing.

7SL RNA, a component of the signal recognition particle (SRP), is the common ancestor of Alu RNAs. Alu elements probably originate from duplication of the 7SL RNA gene [14], [15], [16], [17]. 7SL RNA may form partial dsRNAs with Pol II-transcribed antisense Alu RNAs. It also contains stem-loop structures, the important feature of miRNA precursors [17], [18]. Therefore, it is interesting to address whether Dicer can process 7SL RNA.

In this study, we addressed whether there are endogenous Alu-derived siRNAs in HepG2.2.15 cells, and whether 7SL RNA is processed by Dicer. Using Solexa sequencing technology, we demonstrated that although the abundance of Alu-derived small RNAs was reduced in Dicer knockdown cells, their characteristics were totally different from those of classic siRNAs. Interestingly, we found that 7SL RNA can be processed by Dicer, and the small RNAs derived from 7SL RNA were characterized.

## Results

### The Abundance of Alu-derived Small RNAs is Decreased in Dicer Knockdown Cells

**Figure 1:**
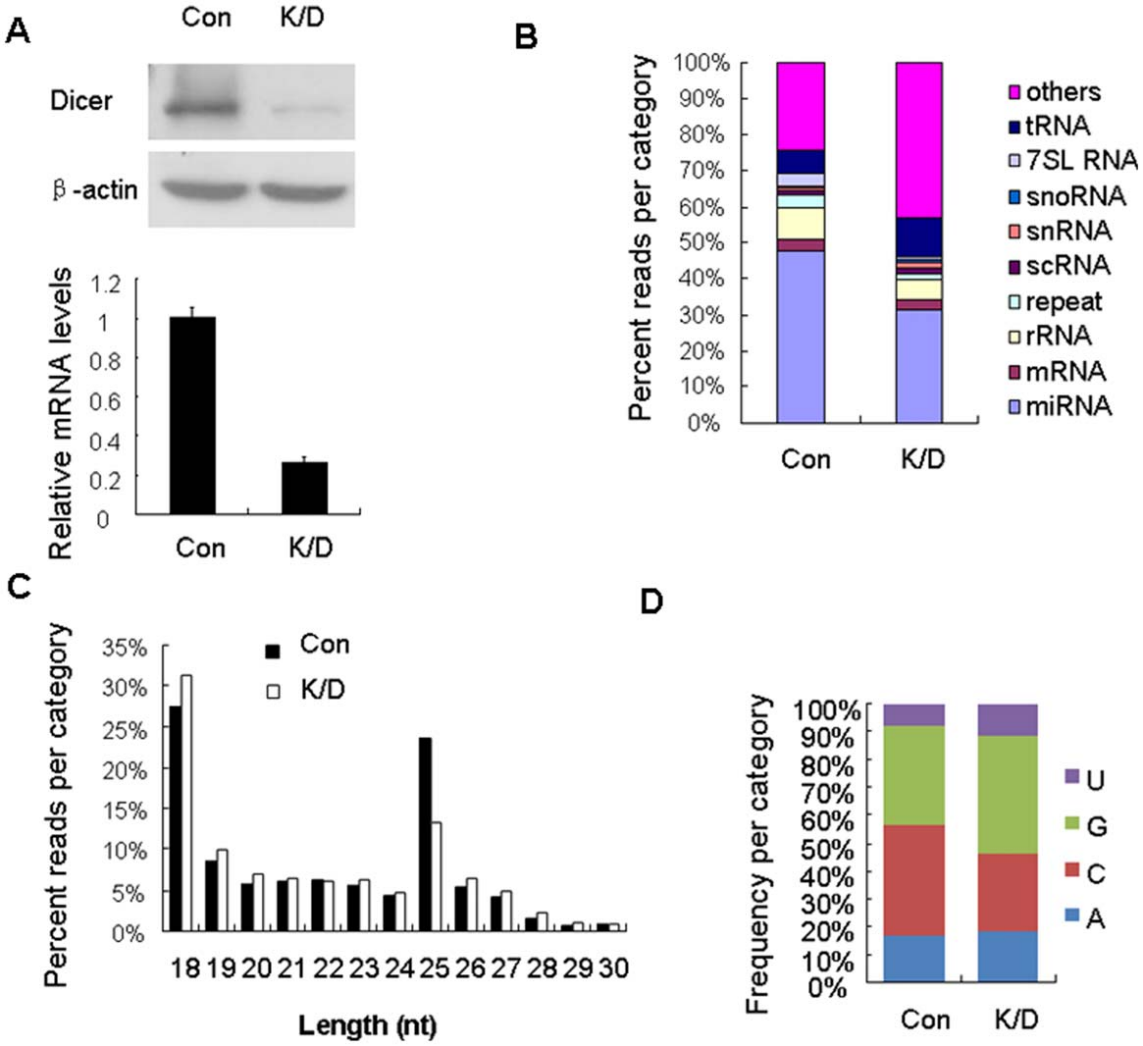
The abundance of Alu-derived small RNAs is decreased in Dicer knockdown HepG2.2.15 cells. (A) Knockdown of Dicer in HepG2.2.15 cells. Top: representative western blot of Dicer performed at 96 h after siRNA transfection, β-actin was used as loading control. Bottom: the relative level of Dicer determined by real-time RT-PCR, data are shown as mean ± SD from three independent experiments. (B) Classification of small RNAs based on UCSC human genome annotations (hg19) in Dicer knockdown (K/D) and the control (Con) cells. (C) Length distribution and (D) First nucleotide bias of Alu-derived small RNAs.

To address whether Alu RNAs are processed by Dicer, we sequenced small RNAs extracted from Dicer knockdown and the control HepG2.2.15 cells using Solexa technology. A total of 14,289,326 genome-matching sequence reads were identified from the control cells, and 14,623,845 from Dicer knockdown cells. The majority (48%) of small RNAs in the control cells were derived from annotated miRNA loci, and as expected, the fraction of miRNA was diminished to 32% in Dicer knockdown cells (Fig. 1B).

Alu-derived small RNAs were identified in our sequencing datasets using BLAST and SOAP2 software [19], [20]. We found that they accounted for 0.6% of the total cellular small RNAs or 1.3% of total miRNAs, and there was a 2.4-fold reduction upon Dicer knockdown (Table 1 and Table S1). Dicer-processed products are usually 21–23 nt in length and disproportionately begin with uridine (U) [21]. However, Alu-derived small RNAs showed length distribution peaks at 18 nt and 25 nt (Fig. 1C), and their first nucleotide was in favor of guanine (G) and cytosine (C) (Fig. 1D).

**Table 1:** Reads and percentage of Alu- and 7SL RNA-derived small RNAs.

	Total small RNAs	Alu-derived small RNAs	7SL RNA-derived small RNAs
Con	14,289,326	88,559 (0.62%)	447,622 (3.1%)
K/D	14,623,845	37,582 (0.26%)	134,136 (0.92%)

### 7SL RNA is Processed by Dicer into Fragments of Different Lengths

**Figure 2:**
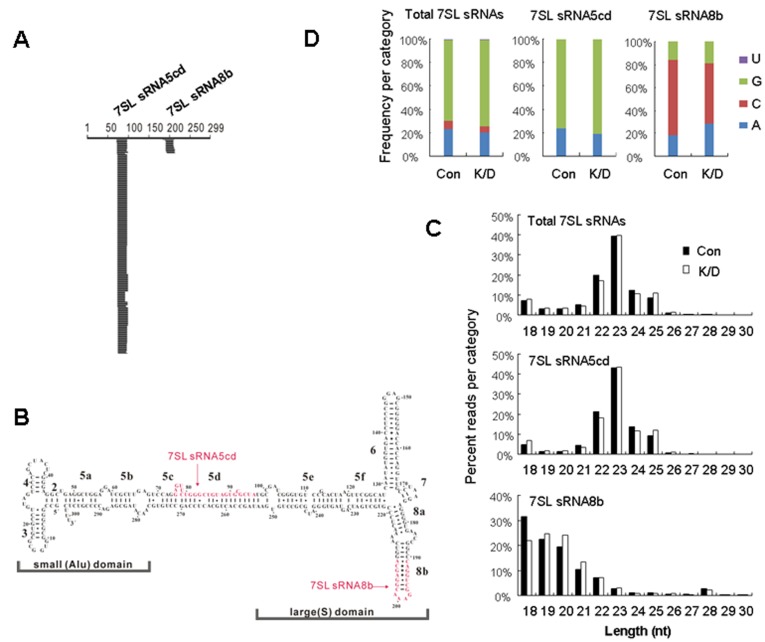
Characterization of 7SL RNA-derived small RNAs. (A) Diagram of 7SL RNA-derived small RNAs in the control cells, the abundance and the length of 7SL RNA-derived small RNAs are drawn as a function of their positions along 7SL RNA. The horizontal line represents the position of small RNAs along the 7SL RNA sequence, the vertical length represents the abundance of small RNAs. (B) Secondary structure of human 7SL RNA. Sequences and positions of 7SL sRNA5cd and 7SL sRNA8b are marked with red color. (C) Length distribution and (D) First nucleotide bias of 7SL RNA-derived small RNAs in Dicer knockdown (K/D) and the control (Con) cells.

7SL RNA, the evolutionary precursor of Alu, is a highly structured RNA containing partial double-stranded regions [17]. We then addressed whether Dicer can process 7SL RNA. The sequences of small RNAs were aligned to the human 7SL RNA sequence using BLAST and SOAP2 software [19], [20]. We found that 7SL RNA-derived small RNAs were highly abundant (accounting for 3.1% of the total cellular small RNAs) in the control cells, and the abundance dropped about 3.4 folds in Dicer knockdown cells (Fig. 1B, Table 1 and Table S2). Moreover, nearly all 7SL RNA-derived small RNAs were from the positive strand (Table S2). Further detailed analysis indicated that most of them were mapped to two sites along the 7SL RNA sequence, and they were classified as 7SL RNA-derived small RNAs-5cd (7SL sRNA5cd) and 7SL RNA-derived small RNAs-8b (7SL sRNA8b), respectively (Figs. 2A and 2B). The characteristics of 7SL RNA-derived small RNAs were in sharp contrast to those of the classic Dicer-processed products. As shown in Figure 2C and Table S2, the length distribution of total 7SL RNA-derived small RNAs and 7SL sRNA5cd showed peak at 23 nt, while the length distribution peak of 7SL sRNA8b was at 18 nt. In addition, the first nucleotide of 7SL RNA-derived small RNAs was in favor of G or C instead of U (Fig. 2D).

**Figure 3:**
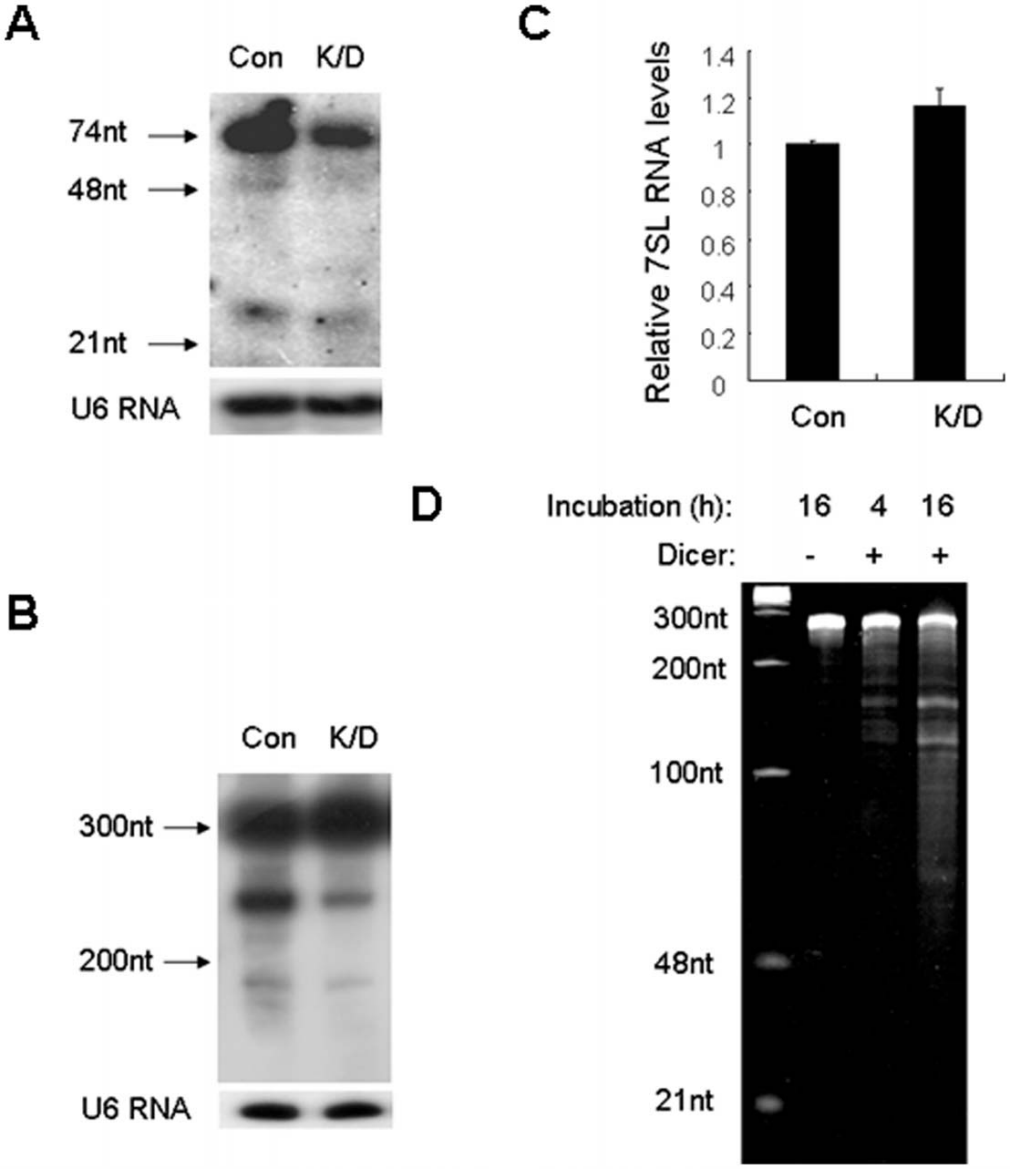
Dicer-dependent processing of 7SL RNA. HepG2.2.15 cells were transfected twice with siDCR or siCon. (A&B) Representative northern blot of 7SL sRNA5cd (A) and the full-length 7SL RNA (B), U6 was used as loading control. (C) The relative level of 7SL RNA determined by real-time RT-PCR, data are shown as mean ± SD from three independent experiments. (D) 12% polyacrylamide gel electrophoresis of 7SL RNA digested by the recombinant human Dicer protein.

To validate Dicer-dependent biogenesis of 7SL RNA-derived small RNAs, we performed northern blotting analysis. As shown in Figures 3A and 3B, a probe complementary to 7SL sRNA5cd detected the 299 nt full-length 7SL RNA, as well as smaller bands of different lengths (including the ∼23 nt small RNAs). The full-length 7SL RNA was much more abundant than the small RNA fragments, implying that only a minor portion of the cellular 7SL RNA pool is processed into small RNAs. Although the level of full-length 7SL RNA was not obviously increased when Dicer was knocked down (Figs. 3B and 3C), the abundance of small RNA fragments was significantly reduced (Figs. 1B, 3A, S1, Table 1 and Table S2). To further confirm that Dicer is able to cleave 7SL RNA, we performed *in vitro* cleavage assay using the recombinant human Dicer protein. As shown in Figure 3D, the synthetic 7SL RNA was cleaved by Dicer into fragments of different lengths. As a negative control, the synthetic LacZ RNA was not processed by Dicer (Fig. S2). Taken together, our data indicate that 7SL RNA is indeed processed by Dicer.

### 7SL RNA-derived Small RNAs do not Function Like miRNAs

**Figure 4:**
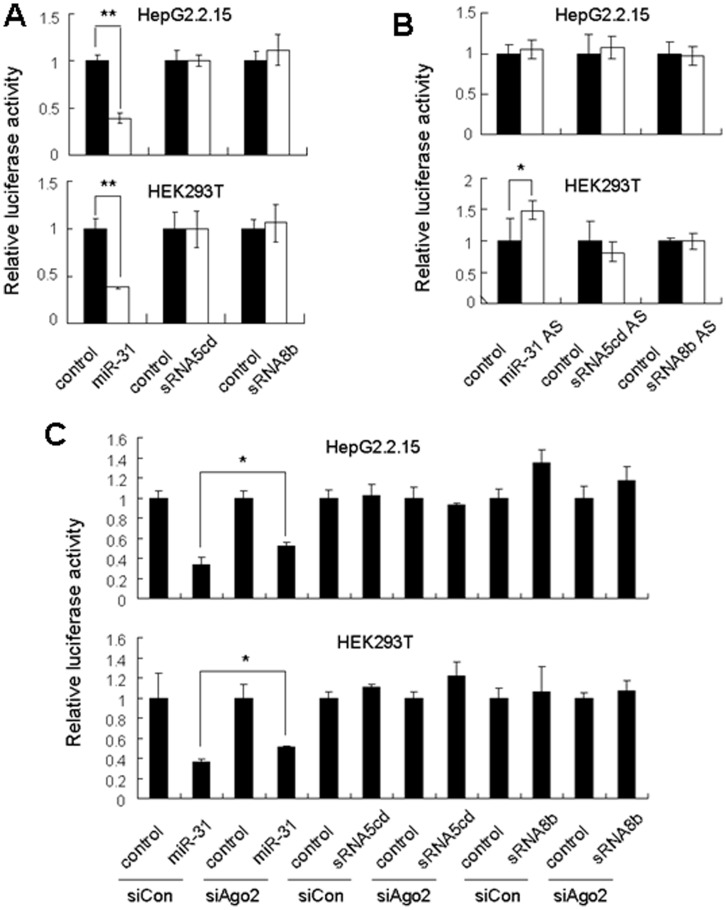
7SL sRNA5cd and 7SL sRNA8b do not function like miRNAs. (A) 7SL sRNA5cd or 7SL sRNA8b was co-transfected with the corresponding luciferase reporter into HEK293T and HepG2.2.15 cells, the luciferase activity was measured 24 h after transfection. (B) The luciferase reporter was co-transfected with the 2′-OMe antisense inhibitor (AS) against 7SL sRNA5cd or 7SL sRNA8b into HEK293T and HepG2.2.15 cells, the luciferase activity assay was performed 24 h after transfection. (*C*) 7SL sRNA5cd or 7SL sRNA8b was co-transfected with the corresponding luciferase reporter into cells that were pretreated with siCon or siAgo2 for 60 h. Luciferase activity was determined 24 h after the co-transfection. Firefly luciferase activity was normalized to Renilla luciferase activity. MiR-31 was used as a positive control. **P<0.01, *P<0.05.

Small RNAs derived from longer non-coding RNAs, such as tRNAs or snoRNAs, have been shown to function like miRNAs [22], [23]. To investigate whether 7SL RNA-derived small RNAs have miRNA like function, we generated luciferase reporters for 7SL sRNA5cd and 7SL sRNA8b and performed luciferase assay in HepG2.2.15 and HEK293T cells. The luciferase activity was not altered when the reporter was co-transfected with the corresponding synthetic small RNA (Fig. 4A), nor was it significantly changed when the endogenous 7SL RNA-derived small RNAs were inhibited by the corresponding 2′-O-methylated (2′-OMe) antisense inhibitor (Fig. 4B). In addition, knockdown of Ago2 (Fig. S3), which is essential for miRNA-mediated translational inhibition [24], did not increase the luciferase activity (Fig. 4C). MiR-31 was used as a positive control. Co-transfection experiments showed that miR-31 decreased the luciferase activity of pGL-LATS2, a reporter construct containing miR-31 binding site downstream the luciferase coding sequence [25] (Fig. 4A). Inhibition of miR-31 by its 2′-OMe antisense inhibitor (miR-31 AS) in HEK293T cells led to an increase in the luciferase activity (Fig. 4B). However, due to the fact that miR-31 was not expressed in HepG2.2.15 cells (data not shown), the effect of miR-31 AS on pGL-LATS2 luciferase activity in this cell line was not significant (Fig. 4B). These results suggest that 7SL RNA-derived small RNAs do not function like miRNAs.

MiRNA is incorporated into the RNA-Induced Silencing Complex (RISC) with Argonaute proteins to regulate gene expression [22], [23], [24]. To address whether 7SL RNA-derived small RNAs are loaded into RISC, we performed RNA immunoprecipitation experiment using anti-Ago2 antibody. As a positive control, we showed that miR-17 was co-immunoprecipitated with Ago2 protein. However, RT-PCR analysis indicated that 7SL sRNA5cd was not associated with Ago2 protein (Fig. S4B), further suggesting that 7SL RNA-derived small RNAs do not function like miRNAs.

### 7SL RNA-derived Small RNAs do not Regulate the Expression of 7SL RNA

**Figure 5:**
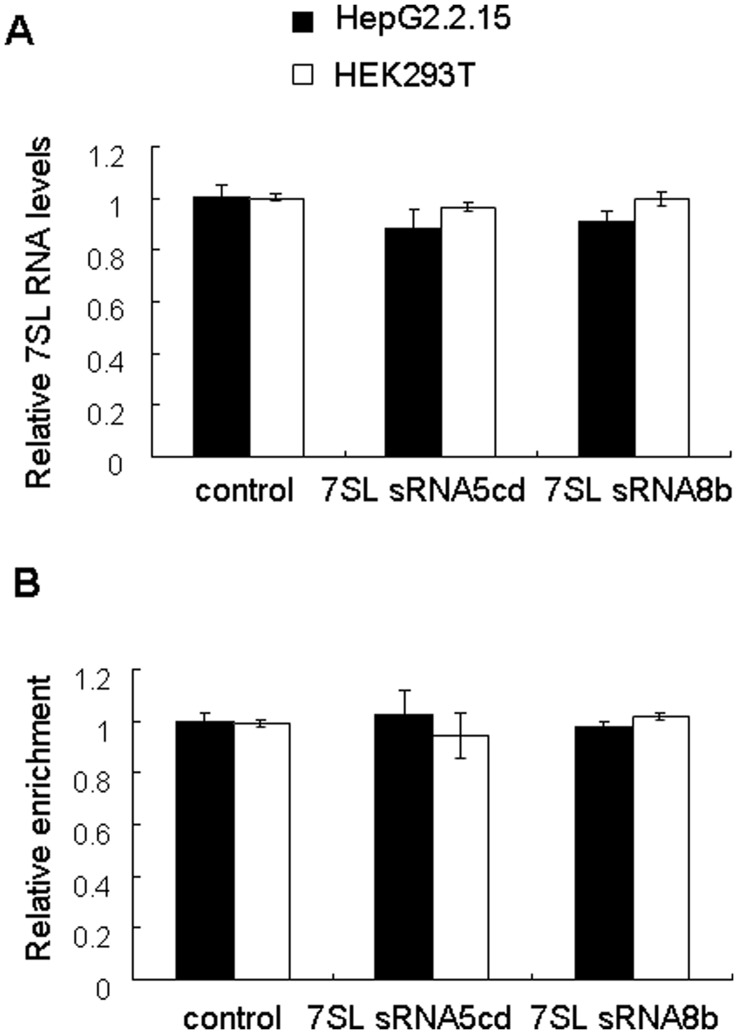
7SL RNA-derived small RNAs do not epigenetically regulate the expression of 7SL RNA. HEK293T and HepG2.2.15 cells were transfected with 7SL sRNA5cd, 7SL sRNA8b or the control small RNA. (A) Real-time RT-PCR was performed at 48 h after transfection to quantify the level of 7SL RNA, *GAPDH* was used as an internal control. Data represent mean ± SD from three independent experiments. (B) ChIP analysis was performed to detect the levels of histone H3 acetylation at 7SL RNA locus. Data represent mean ± SD from three independent experiments.

Growing evidence has suggested that small RNAs are implicated in epigenetic regulation of gene expression [26], [27]. To address whether 7SL RNA-derived small RNAs can epigenetically regulate 7SL RNA expression, we transfected synthetic 7SL sRNA5cd or 7SL sRNA8b into HEK293T and HepG2.2.15 cells. Real-time RT-PCR results indicated that neither 7SL sRNA5cd nor 7SL sRNA8b transfection caused significant change of 7SL RNA level (Fig. 5A). In addition, ChIP analysis indicated that histone H3 acetylation level at 7SL RNA locus was not changed upon transfection of 7SL sRNA5cd or 7SL sRNA8b (Fig. 5B).

## Discussion

We have demonstrated that the abundance of 7SL RNA-derived small RNAs was decreased in Dicer knockdown cells (Figs. 1B, 3A, S1, Table 1 and Table S2), and that the recombinant human Dicer protein can process 7SL RNA *in vitro* (Fig. 3D). These results suggest that 7SL RNA-derived small RNAs are Dicer-processed products. However, 7SL sRNA5cd and 7SL sRNA8b, two major 7SL RNA-derived small RNAs, showed length distribution peaks at 23 nt and 18 nt, respectively (Fig. 2C). In addition, their first nucleotide had a bias in favor of G or C, instead of U (Fig. 2D). These characteristics are different from those of miRNAs and siRNAs, the classic Dicer-processed products. In consistent with these characteristics, 7SL RNA-derived small RNAs did not function like miRNAs (Fig. 4), neither did they regulate the expression of 7SL RNA (Fig. 5).

7SL RNA was first detected in Rous sarcoma virus particles [28], and later identified as a stable component of the SRP complex [16], [17]. Recently, it has been reported that 7SL RNA is packaged into HIV-1 virions, and an endoribonucleolytic fragment of 7SL RNA (termed 7SLrem) was found in HIV-1 virions and the minimal virus-like particles [29], [30], [31], [32], [33]. The ends of 7SLrem map to bulges in the secondary structure of the full-length 7SL RNA where nucleotides remain unpaired, implying that the full-length 7SL RNA is processed into 7SLrem by a single-stranded endonuclease [30]. We show here that in addition to the (as yet unidentified) single-stranded endonuclease, Dicer, a double-stranded endonuclease, is also involved in the processing of 7SL RNA. It is worth to note that Dicer only processes a minor portion of the cellular 7SL RNA pool into small RNAs. Therefore, the level of full-length 7SL RNA was only slightly increased in Dicer knockdown cells, even though the abundance of small RNA fragments was significantly reduced (Figs. 3A–C, S1, Table 1 and Table S2). This result is in consistent with that of Kaneko and colleagues, who showed that decreased Dicer expression did not affect the level of full-length 7SL RNA [7].

Dicer dependent SINE/B1-derived endogenous siRNAs have been identified [9], [10], [11], [12], [13], and it has been demonstrated that decreased Dicer expression leads to Alu RNAs accumulation in human retinal pigmented epithelium cells [7]. The SINE/B1-derived small RNAs exhibit two peaks (22 nt and 27 nt) in mouse embryos and blastocysts [13], while Alu-derived small RNAs show two peaks at 18 nt and 25 nt in HepG2.2.15 cells (Fig. 1C). In addition, Alu-derived small RNAs only account for 0.6% of the total cellular small RNAs, and are much less abundant (16-fold) than miR-21, the highest miRNA in HepG2.2.15 cells. Consider the fact that Alu RNAs are highly abundant in human cells [1], [2], [3], the level of Alu-derived small RNAs is extremely low. Moreover, unlike the classic Dicer processed small RNAs, the first nucleotide of Alu-derived small RNAs shows a bias in favor of G and C instead of U (Fig. 1D). Taken together, although we showed that the abundance of Alu-derived small RNAs was reduced in Dicer knockdown HepG2.2.15 cells, further investigation is needed to address whether Dicer can cleave the endogenous Alu RNAs into siRNAs.

In summary, we found that Dicer can process 7SL RNA into fragments of different lengths. The molecular functions of these 7SL RNA-derived small RNAs are still under investigation.

## Materials and Methods

### Cell Culture and Transfection

Human embryonic kidney cells (HEK293T from ATCC) and HepG2.2.15 cells (a hepatoma cell line that constitutively expresses HBV [34]) were cultured in RPMI 1640 medium supplemented with 10% FBS. All cultures were maintained at 37°C in a moist atmosphere containing 5% CO_2_. Small RNAs were obtained from Invitrogen (Shanghai, China). Dicer knockdown was performed as described previously [35]. Plasmid transfection was performed using Lipofectamine 2000 (Invitrogen, Grand Island) and RNA transfection was performed using siPORT NeoFX (Ambion, Austin) according to the manufacturer’s instructions. The interference efficiency was evaluated by real-time RT-PCR and western blotting.

### Western Blotting

Cells were lysed in RIPA buffer, equal amounts of denatured total protein were subjected to SDS-PAGE and then transferred to polyvinylidene fluoride membranes (Millipore, Bedford). Membranes were incubated with primary antibody, followed by horseradish peroxidase-linked secondary antibody and detected with ECL plus reagents (Millipore). The primary antibodies include: anti-Dicer (Abcam, Cambridge, USA), anti-Ago2 (Cell Signaling Technology, Danvers) and anti-β-actin (Boster, Wuhan).

### Small RNA Sequencing and Bioinformatics Analysis

Small RNAs ranging from 16 to 30 nt were gel-purified, and those with 5′-phosphate and 3′-hydroxyl group were ligated with the 3′- and 5′-adaptors. The ligation products were then gel-purified, reversely transcribed, and PCR-amplified. The PCR fragments were then sequenced using Illumina GAII platform. Low quality sequencing reads were discarded, and the adaptor sequences were removed before analysis of small RNA sequences. The sequences were mapped to the reference human genome (hg19) using SOAP2 program with at most two mismatches. For annotation, small RNA sequences were aligned to miRBase15.0, Rfam 9.1 and GeneBank using BLAST and SOAP2 software. Alu or 7SL RNA-derived small RNAs were identified by mapping the small RNA sequences to the human Alu sequences extracted from the TranspoGene database (http://transpogene.tau.ac.il/) and the human 7SL RNA gene sequence (NR_002715.1), respectively.

### Northern Blotting

HepG2.2.15 cells were transfected with siDCR or siCon, and harvested 96 h after transfection. Total RNAs were prepared using TRIzol reagent (Invitrogen), and small RNAs were extracted using the E.Z.N.A.® miRNA Isolation Kit (Omega, Norcross).

To detect the full-length 7SL RNA, 10 µg total RNAs were separated by electrophoresis in an 8% polyacrylamide gel and transferred to a nylon membrane. The membrane was hybridized with [γ-^32^P]ATP labeled oligonucleotides complementary to 7SL sRNA5cd. U6 was used as loading control.

To detect small RNAs derived from 7SL RNA, 30 µg small RNAs were separated by electrophoresis in a 15% polyacrylamide gel and transferred to a nylon membrane. The membrane was hybridized with [γ-^32^P]ATP labeled oligonucleotides complementary to 7SL sRNA5cd. U6 was used as loading control.

### Plasmids Construction

The pMD-7SL plasmid was generated by inserting the following sequence into the pMD18-T vector (TaKaRa, Dalian), and the specific sequences are marked with different font formats as indicated.

AAGCTT *AATTATAATACGACTCACTATAGGGAGA* **CCGCGCCCGGCCTGATGAGTCCGTGAGGACGAAACGGTACCCGGTACCGTC**GCCGGGCGCGGTGGCGCGTGCCTGTAGTCCCAGCTACTCGGGAGGCTGAGGCTGGAGGATCGCTTGAGTCCAGGAGTTCTGGGCTGTAGTGCGCTATGCCGATCGGGTGTCCGCACTAAGTTCGGCATCAATATGGTGACCTCCCGGGAGCGGGGGACCACCAGGTTGCCTAAGGAGGGGTGAACCGGCCCAGGTCGGAAACGGAGCAGGTCAAAACTCCCGTGCTGATCAGTAGTGGGATCGCGCCTGTGAATAGCCACTGCACTCCAGCCTGGGCAACATAGCGAGACCCCGTCTCTCATTGC .

(Underline: restriction enzyme recognition sequences; *Italic*: T7 promoter sequence; **Bold**: ribozyme sequence; Regular: 7SL RNA sequence).

MiR-31 luciferase reporter pGL-LATS2 was generated by inserting the 3′-UTR of LATS2, which contains miR-31 binding site, into the pGL3-control vector (Promega, Madison) at the XbaI site immediately downstream the stop codon of firefly luciferase. The following primers were used to amplify the 3′-UTR of LATS2∶5′-CTAGTCTAGAATGATGTGGCTGTGATTTCC-3′ and 5′-CTAGTCTAGAGGTAAGTAGTAGGGTCAGAGGTAT-3′.

Luciferase reporters for 7SL RNA-derived small RNAs (pGL5cd and pGL8b) were constructed by inserting the annealed oligos containing the perfectly complementary binding site for 7SL sRNA5cd or 7SL sRNA8b into the pGL3-control vector at the XbaI site. Oligo sequences are as follows: pGL5cd, 5′-CTAGGGAGTTCTGGGCTGTAGTGCGCTATGCCGAT-3′ and 5′-CTAGATCGGCATAGCGCACTACAGCCCAGAACTCC-3′; pGL8b, 5′-CTAGTGAACCGGCCCAGGTCGGAAACGGAGCAGGTCAAAACTCC-3′ and 5′-CTAGGGAGTTTTGACCTGCTCCGTTTCCGACCTGGGCCGGTTCA-3′.

### RNA Preparation and in vitro Dicer Cleavage Assay

The 299 nt full-length 7SL RNA was transcribed *in vitro* as described previously [36], and gel purified using 7 M urea denaturing PAGE. The *in vitro* transcription template was PCR-amplified from pMD-7SL with the following primers: 5′-AAGCTTAATTATAATACGACTCACTATAG-3′ and 5′-AGAGACGGGGTCTCGCTATGTTG-3′. The LacZ control template was PCR-amplified using the following primers: 5′-AATTATAATACGACTCACTATAGGGAGACCGCGCCCGGCCTGATGAGTCCGTGAGGACGAAACGGTACCCGGTACCGTCGACGTCTCGTTGCTGCATAA-3′ and 5′-ACAGTTCCGGATTTTCAACG-3′. For *in vitro* Dicer cleavage assay, gel-purified 7SL RNA and LacZ RNA was incubated with the recombinant human Dicer protein (Invitrogen) at 37°C.

### Quantitative Real-time RT-PCR

RNA was prepared using TRIzol reagent and treated by RNase-free DNase I (Fermentas, Glen Burnie) for 30 min. The DNA-free RNA was reversely transcribed using the M-MLV reverse transcription kit (Promega) according to the manufacturer’s instructions. Sample prepared without reverse transcription served as a negative control. SYBR green real-time PCR was performed with the ABI PRISM 7300 Sequence Detection system (Applied Biosystems). All samples were normalized to *GAPDH*. Primer sequences for Dicer and *GAPDH* are as described previously [35]. Other primer sequences are as follows: *Ago2*, 5′-TGCCTTCAAGCCTCCACCTA-3′ and 5′-GTGTTCCACGATTTCCCTGTT-3′; 7SL RNA, 5′-GGAGTTCTGGGCTGTAGTGC-3′ and 5′-ATCAGCACGGGAGTTTTGAC-3′.

### Luciferase Reporter Assays

Cells were co-transfected with 0.4 µg firefly luciferase reporter vector (pGL5cd, pGL8b or pGL-LATS2), 0.02 µg Renilla luciferase control vector (pRL-CMV, Promega), and different small RNAs at a final concentration of 100 nM, using Lipofectamine 2000 in the 24-well plates. Luciferase assays were performed 24 h after transfection using the dual-luciferase reporter assay system (Promega). Small RNAs, including miR-31, 7SL sRNA5cd and 7SL sRNA8b, and their 2′-OMe antisense inhibitors were obtained from Invitrogen. For *Ago2* knockdown experiments, cells were transfected twice with siAgo2 (target sequence: GCACGGAAGTCCATCTGAA) or siCon 60 h prior to co-transfection of the reporter plasmid and small RNA, and luciferase assays were performed 24 h after the co-transfection. Firefly luciferase activity was normalized to Renilla luciferase activity.

### Chromatin Immunoprecipitation

Chromatin immunoprecipitation assay was carried out using Chromatin Immunoprecipitation Assay Kit (Millipore) according to the manufacturer’s instructions with anti-H3Ac antibody (Millipore) and the mouse IgG control (Active Motif, Carlsbad). The precipitated DNA was recovered, and quantified by real-time PCR.

### RNA Immunoprecipitation and RT-PCR

Immunoprecipitation of Ago2 binding RNA was carried out as previously described [37] with minor modifications. Briefly, HepG2.2.15 cell extract was prepared with 2X Ago2 IP lysis buffer [40 mM Tris (pH7.5), 300 mM NaCl, 3 mM MgCl_2_, 0.5% NP-40, 1 mM PMSF and protease inhibitors (Sigma, St. Louis)], immunoprecipitated with an anti-Ago2 antibody (Cell Signaling Technology) and then bound to Protein-G Sepharose (GE Healthcare, Shanghai) beads. The beads were washed extensively with Ago2 IP washing buffer [50 mM Tris (pH7.5), 300 mM NaCl, 5 mM MgCl_2_ and 0.1% NP-40] before elution with 0.1 M glycine (pH2.3). Ago2-bound RNAs were then isolated using Trizol LS reagent (Invitrogen). To detect 7SL sRNA5cd, the eluted RNA was poly A tailed using a Poly (A) Polymerase Tailing Kit (EPICENTRE, Madison), reversely transcribed using RT primer (ATTCTAGAGGCCGAGGCGGCCGACATGTTTTTTTTTTTTTTTTTTTTTTTTTTTTTTVN) and PCR amplified using the following primers: GAGTTCTGGGCTGTAGTGCGCTA and ATTCTAGAGGCCGAGGCGGCC, which only detected 7SL sRNA5cd but not the full-length RNA (Fig S3A). MiR-17 was detected using the miR-17 RT-PCR kit from RiboBio (Guangzhou).

## Supporting Information

Figure S1 **Quantification of small RNA northern blotting.** 7SL sRNA5cd was represented by the 23 nt band.
(TIF)

Figure S2 **Dicer cannot cleave the control RNA.** 12% polyacrylamide gel electrophoresis of LacZ RNA digested by the recombinant human Dicer protein.
(TIF)

Figure S3 **Knockdown of Ago2 in HepG2.2.15 and HEK293T cells.** (A&C) representative western blot of Ago2, β-actin was used as loading control. (B&D) The relative levels of Ago2 mRNA determined by real-time RT-PCR. (A&B) Hep2.2.15 cells, (C&D) HEK293T cells.
(TIF)

Figure S4 **7SL sRNA5cd is not associated with Ago2 protein.** (A) RT-PCR can specifically detect 7SL sRNA5cd but not full-length 7SL RNA. (B) RNA immnuoprecipitation experiment indicated that 7SL sRNA5cd was not associated with Ago2 protein, as a positive control, miR-17 was co-immunoprecipitated with Ago2 protein.
(TIF)

Table S1 **Alu-derived small RNAs.**
(XLS)

Table S2 **7SL RNA-derived small RNAs.**
(XLS)
