# Supplementary figures and images for: Dicer-Dependent Biogenesis of Small RNAs Derived from 7SL RNA
Source: PLoS One. 2012 Jul 12;7(7):e40705. doi: 10.1371/journal.pone.0040705 (PMC3395682; doi:10.1371/journal.pone.0040705)

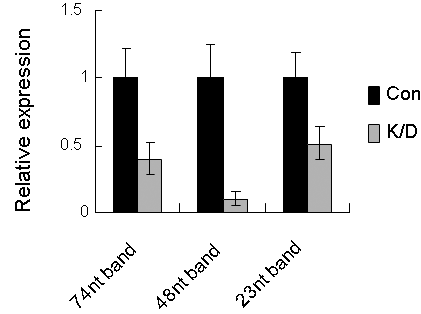

Supplement: Figure S1 — Quantification of small RNA northern blotting. 7SL sRNA5cd was represented by the 23 nt band. (TIF) [file pone.0040705.s001.tif]

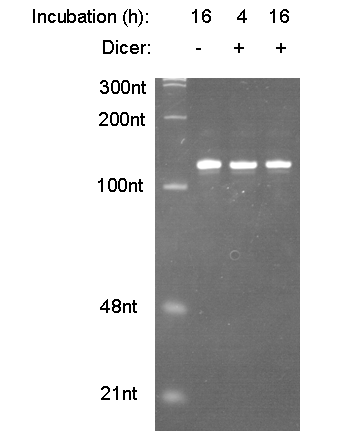

Supplement: Figure S2 — Dicer cannot cleave the control RNA. 12% polyacrylamide gel electrophoresis of LacZ RNA digested by the recombinant human Dicer protein. (TIF) [file pone.0040705.s002.tif]

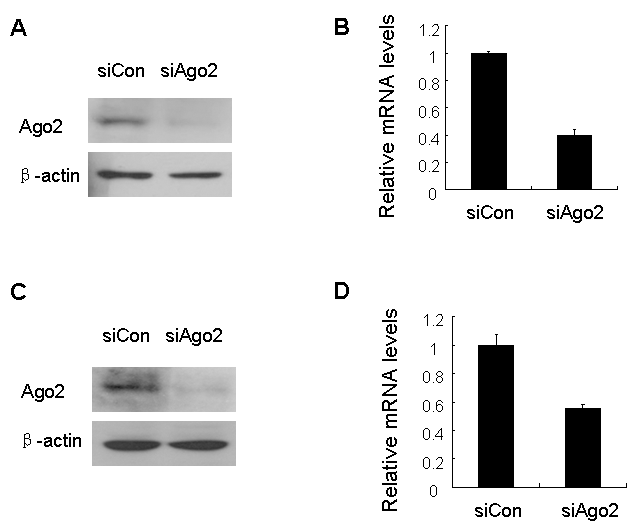

Supplement: Figure S3 — Knockdown of Ago2 in HepG2.2.15 and HEK293T cells. (A&C) representative western blot of Ago2, β-actin was used as loading control. (B&D) The relative levels of Ago2 mRNA determined by real-time RT-PCR. (A&B) Hep2.2.15 cells, (C&D) HEK293T cells. (TIF) [file pone.0040705.s003.tif]

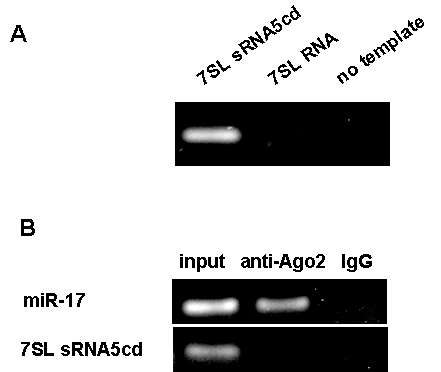

Supplement: Figure S4 — 7SL sRNA5cd is not associated with Ago2 protein. (A) RT-PCR can specifically detect 7SL sRNA5cd but not full-length 7SL RNA. (B) RNA immnuoprecipitation experiment indicated that 7SL sRNA5cd was not associated with Ago2 protein, as a positive control, miR-17 was co-immunoprecipitated with Ago2 protein. (TIF) [file pone.0040705.s004.tif]
